# An increased risk of epithelial ovarian cancer in Taiwanese women with a new surgico-pathological diagnosis of endometriosis

**DOI:** 10.1186/1471-2407-14-831

**Published:** 2014-11-18

**Authors:** Kuan-Chin Wang, Wen-Hsun Chang, Wen-Ling Lee, Nicole Huang, Hsin-Yi Huang, Ming-Shyen Yen, Chao-Yu Guo, Peng-Hui Wang

**Affiliations:** Department of Nursing, Oriental Institute of Technology, New Taipei City, Taiwan; Department of Nursing, Taipei Veterans General Hospital, Taipei, Taiwan; Department of Nursing, National Yang-Ming University School of Nursing, Taipei, Taiwan; Institute of Public Health, and Institute of Hospital and Health Care Administration, National Yang-Ming University, Taipei, Taiwan; Division of Gynecology, Taipei Veterans General Hospital, Taipei, Taiwan; Department of Medicine, Cheng-Hsin General Hospital, Taipei, Taiwan; Department of Obstetrics and Gynecology, National Yang-Ming University, Taipei, Taiwan; Biostatics Task Force, Taipei Veterans General Hospital, Taipei, Taiwan; Department of Medical Research, China Medical University Hospital, Taichung, Taiwan; Immunology Center, Taipei Veterans General Hospital, Taipei, Taiwan; Division of Gynecology, Taipei Veterans General Hospital and Department of Obstetrics and Gynecology, National Yang-Ming University School of Medicine, 201, Section 2, Shih-Pai Road, Taipei, 112 Taiwan

**Keywords:** Cohort study, Endometriosis, Epidemiology, Epithelial ovarian cancer

## Abstract

**Background:**

Epidemiological evidence of relationships between endometriosis and epithelial ovarian cancer (EOC) has been obtained mainly from Western countries. Our goal was to determine the risk of EOC due to endometriosis in Taiwanese women.

**Methods:**

A retrospective cohort study was performed by linking to the National Health Insurance Research Database (NHIRD) of Taiwan. A total of 5,945 women with a new surgico-pathological diagnosis of endometriosis from 2000 to 2010 and 23,780 multivariable-matched controls (1:4) were selected. The Cox regression model adjusted for potential confounders was used to assess the risk of EOC due to endometriosis.

**Results:**

The EOC incidence rate (IR) of the women with and without endometriosis was 11.64 and 2.66 per 10,000 person-years, contributing to a crude hazard ratio (HR) of 4.48 (95% confidence interval [CI] 2.84-7.06), and HR after adjustment for all confounders (adjusted HR) of 5.62 (95% CI 3.46-9.14); the risk was higher in clear-cell carcinoma subtypes (adjusted HR 7.36, 95% CI 1.91-28.33). The EOC IR of women with endometriosis consistently increased with increasing age, ranging from 4.99 (<30 years) to 35.81 (≥50 years) per 10,000 person-years, contributing to a progressively increased risk of EOC (crude HRs ranging from 2.80 to 6.74 and adjusted HRs ranging from 3.34 to 9.63) compared to age-matched women without endometriosis, whose EOC IR also increased with age. The older women (≥50 years) with endometriosis had a risk of EOC that was higher than both the age-matched women without endometriosis (adjusted HR 9.63, 95% CI 3.27-28.37) and the youngest women (<30 years) with endometriosis (adjusted HR 4.97, 95% CI 1.03-24.09).

**Conclusions:**

These significant findings corroborate the previously reported association between endometriosis and increased risk of EOC. Since the risk of EOC in women with a new surgico-pathological diagnosis of endometriosis constantly increased with age and this increased risk of EOC was more significant in women aged ≥50 years, active and intensive surgical intervention should be taken into consideration for older women with endometriosis.

## Background

Many epidemiologic studies have supported the finding that women with endometriosis may have an increased risk of developing and/or being associated with epithelial ovarian cancers (EOCs) [1–23]. However, nearly all evidence has been obtained from Western countries [3–25]. A recent meta-analysis by Kim and colleagues investigating the impact of endometriosis on the risk and prognosis of ovarian cancer concluded that endometriosis is strongly associated with an increased of ovarian cancer but endometriosis may not affect disease progression after the onset of ovarian cancer [25]. However, the risk ratio (RR) was only 1.27 (95% confidence interval [CI] =1.21-1.32) in case–control or two-arm cohort studies and the standard incidence ratio (SIR) was 1.80 (95% CI 1.28-2.53), respectively [25]. The RR of “endometriosis-associated EOC”, including endometrioid and clear-cell subtypes of EOC in women with endometriosis, was 1.76 (95% CI 1.55-2.00) and 2.61 (95% CI 2.23-3.05), respectively [25].

Except the strong association between the clear-cell subtype and EOC, all others seemed to be well under the level that supports causality. Why did the data show only a weak association between endometriosis and EOC? Although it is difficult to respond to the above question, a possible reason might be the heterogeneity within the newly defined population, since clinical heterogeneity, when present, has implications for the design of research studies [26–28]. For example, Buis and colleagues found that an increased risk was found in women with endometriosis when the definition of endometriosis was based on self-report, medical records information at subfertility treatment and/or a nationwide pathology database (HR 8.2, 95% CI 3.1-21.6) [22]. The risk was especially high in women with pathologically-confirmed endometriosis after subfertility treatment, with a HR of 12.4 (95% CI 2.8-54.2) [22]. In addition, previous studies might have been influenced by any one or more of many factors, such as age, obstetric and gynecologic history (nulliparity, menstrual cycles, infertility status, pelvic inflammatory disease [PID], or hysterectomy history, the use of pills and tubal ligation) and many chronic illnesses -- such as cardiovascular disease (CVD), diabetes mellitus (DM), chronic liver disease (CLD), and rheumatic disease (RD), that might contribute to the estimation of cancer risk [14, 20]. And, to avoid surveillance bias for cancer, which may shorten the time to cancer diagnosis and cause over-estimation of risks, most studies excluded synchronous cases (defined as having a time interval between the detection of endometriosis and EOC of 6 to 12 months), resulting in an under-estimation of the association. The aim of this study was to investigate whether endometriosis was really associated with EOC after adjusting the above-mentioned factors. In order to achieve our aim, we conducted a large-scale, nationwide, controlled cohort study.

## Methods

The source population consisted of nearly the entire population of Taiwan (23 million inhabitants) and the data was that of the research database of the Taiwanese National Health Insurance (NHI) program from 1996 to 2010. The Longitudinal Health Insurance Database 2000 (LHID 2000) contains 1 million randomly sampled beneficiaries. The data of the sampled subjects in the LHID 2000 are representative of all beneficiaries with regard to age, sex, and insurance cost, which have been described in detail before [29, 30].

This was a retrospective cohort study. According to the written operating procedures, Good Clinical Practice (GCP), and the applicable regulatory requirements, this study projected was approved by the Institutional Review Board of Taipei Veterans General Hospital (Chairman, Professor Shung-Tai Ho, VGHIRB No.: 2012-12-012BC), and the board is organized under, and operates according to International Conference on Harmonisation (ICH)/WHO GCP and the applicable laws and regulations. The National Health Research Institute in Taiwan permitted the access to the data in the National Health Insurance Research Database, and 29,725 women aged between 20 and 51 years were identified. Women without a visit to an obstetrician or gynecologist during the study period were excluded. In order to increase the validity of identifying women with newly diagnosed endometriosis in the administrative data set, only those women with a new surgico-pathological diagnosis of endometriosis (International Classification of Diseases, Ninth Revision, and Clinical Modifications [ICD9-CM] code 617), regardless of clinical diagnosis of endometriosis status, during the period between January 1, 2000 and December 31, 2010 were included among the incident endometriosis women (*n* =5,945).

To validate the surgico-pathological diagnosis of endometriosis, surgical treatments for endometriosis especially limited to the ovary, tube, and peritoneal cavity were also recorded. These included laparoscopy (ICD9-CM codes 54.21) and laparoscopic surgery, such as laparoscopic lysis of peritoneal adhesions (54.51), laparoscopic oophorectomy (65.01), laparoscopic diagnostic procedures related to the ovaries (65.13 and 65.14), laparoscopic local excision or destruction of the ovaries (65.23, 65.24, and 65.25), laparoscopic unilateral oophorectomy (65.31), laparoscopic unilateral salpingo-oophorectomy (65.41), laparoscopic salpingo-oophoroplasty (65.76), and laparoscopic lysis of adhesions to the ovary and fallopian tube (65.81). Exploratory laparotomy, such as lysis of peritoneal adhesions (54.59), oophorectomy (65.0), aspiration biopsy of the ovary (65.11 and 65.12), local excision or destruction of the ovary (65.21, 65.22, and 65.29), unilateral oophorectomy (65.3), unilateral salpingo-oophorectomy (65.49), salpingo-oophoroplasty (65.73), and lysis of adhesions to the ovary and fallopian tube (65.89), was also included. However, to decrease the influence of hysterectomy, bilateral salpingo-oophorectomy, and bilateral oophorectomy on the development of future EOC, women with hysterectomy, except those women with a diagnosis of invasive EOC during the follow-up period, were excluded.

**Figure 1:**
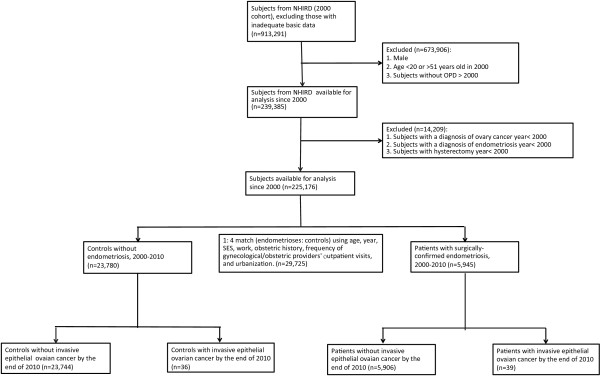
**Cohort flow chart illustrating the inclusion and exclusion of participants in the study.**

Each endometriosis case was matched with 4 female controls by age, index year, obstetric history, frequency of gynecological/obstetric providers’ outpatient visits, contraception methods, socioeconomic status, work and urbanization, which resulted in an overall sample size of 23,780 matched controls without endometriosis (Figure 1). For the women with endometriosis, the index date was the date of a new surgico-pathological diagnosis of endometriosis. For the controls, the index date was the first visit to an obstetric/gynecological provider or admission during the study period.

EOC was initially detected using inpatients with a surgico-pathological diagnosis and validated using the major disease files (ICD-9-CM 183) from the Registry for Catastrophic Illness Patients.

Starting from the cohort index date, the study subjects were followed until hospitalization with EOC or to the end of the study (December 31, 2010), whichever came first, if no EOC had occurred. The specific histologic subtype distribution of EOC in women with and without endometriosis came from the database of the National Cancer Registration System [31]. The histological types used were based on the World Health Organization Classification of Tumors [32], and included serous (8441/3, 8460/3, 8461/3), mucinous (8470/2, 8470/3, 8471/3, 8480/3, 8482/3), endometrioid (8380/3, 8382/3, 8383/3), clear cell (8310/3, 8313/3), malignant Brenner (9000/3), undifferentiated (8020/3, 8021/3), and carcinosarcoma (8950/3, 8980/3, 8981/3). “Endometriosis-associated” EOCs, including clear-cell and endometrioid-cell types, were reported to be highly associated with endometriosis [10, 17, 23–25]. Patients without an EOC event were treated as censored subjects. Dropouts or those who were lost to follow-up were also treated as censored. Basic characteristics are presented as percentages. The incidence of EOC was compared between the women with and without endometriosis using the incidence rate (IR). The χ2 test was used to compare the IR estimates of invasive EOC among subsamples. The Cox proportional hazards model was used to calculate the HR and 95% CI to determine whether newly diagnosed endometriosis is a risk factor for EOC.

In order to handle the issue of matching, the robust sandwich estimate of Lin and Wei [33] for the covariance matrix was used in the Wald tests to test the global null hypothesis and null hypotheses of individual parameters. Variables adjusted in the Cox model were PID, infertility status, CVD, DM, CLD and RD. Statistical analyses were implemented with SAS version 9.3 (SAS Institute Inc., Cary, North Carolina, USA), STATA version 10.0 (STATA Corp, College Station, Texas, USA), and SPSS version 20 (SPSS, Chicago, IL, USA).

## Results

Seventy-five of the total 29,725 women had EOC between 2000 and 2010. The total person-years of follow-up were 168,927, including 33,519 for the women with a surgico-pathological diagnosis of endometriosis and 135,408 for the women without endometriosis. The women with endometriosis had higher rates of comorbid PID, infertility, CVD, DM, CLD, and RD than did the women without endometriosis (all *p* <0.0005) (Table 1).

**Table 1:** **Baseline characteristics of the study subjects**

	Total (***n*** = 29725)		Endometriosis patients (***n*** = 5945)		Controls (***n*** = 23780)		P-value
Person-years	168927		33519		135408		
Variable	n	%	n	%	n	%	
Catastrophic illness							< 0.0001
EOC	75	0.25	39	0.66	36	0.15	
No EOC	29650	99.75	5906	99.34	23744	99.85	
Age*							0.0750
≤ 41	14877	50.05	2914	49.02	11963	50.31	
e> 41	14848	49.95	3031	50.98	11817	49.69	
SES							0.8868
≥ 40000	4579	15.40	911	15.32	3668	15.42	
20000-39999	9790	32.94	1941	32.65	7849	33.01	
< 20000	10236	34.44	2073	34.87	8163	34.33	
Others	5120	17.22	1020	17.16	4100	17.24	
Work							0.5954
Yes	27617	92.91	5514	92.75	22103	92.95	
No	2108	7.09	431	7.25	1677	7.05	
Urbanization							0.9752
Urban	9957	33.50	1991	33.49	7966	33.50	
Suburban	12549	42.22	2504	42.12	10045	42.24	
Rural	7219	24.29	1450	24.39	5769	24.26	
PID							< 0.0001
Yes	13647	45.91	4518	76.00	9129	38.39	
No	16078	54.09	1427	24.00	14651	61.61	
Infertility							< 0.0001
Yes	1094	3.68	608	10.23	486	2.04	
No	28631	96.32	5337	89.77	23294	97.96	
CV disease							< 0.0001
Yes	1133	3.81	290	4.88	843	3.54	
No	28592	96.19	5655	95.12	22937	96.46	
Diabetes mellitus							< 0.0001
Yes	1820	6.12	448	7.54	1372	5.77	
No	27905	93.88	5497	92.46	22408	94.23	
Chronic liver disease							0.0002
Yes	477	1.60	128	2.15	349	1.47	
No	29248	98.40	5817	97.85	23431	98.53	
Rheumatic disease							< 0.0001
Yes	796	2.68	235	3.95	561	2.36	
No	28929	97.32	5710	96.05	23219	97.64	

EOC: invasive epithelial ovarian cancer; SES: socio-economic status; PID: pelvic inflammatory disease; CV disease: cardiovascular disease.

*Age variable was matched by the exact year of age, but the table shows age quartile groups. The median age of women with and without endometriosis was 40.5 and 40.4 years of age, respectively (*p* = 0.3570).

The EOC IR of the women with and without a surgico-pathological diagnosis of endometriosis was 11.64 and 2.66 per 10,000 person-years, respectively, contributing to a crude HR of 4.48 (95% CI 2.84-7.06), and HR after adjustment for confounders (adjusted HR) of 5.62 (95% CI 3.46-9.14) (Table 2).

**Table 2:** **Incidence and crude and adjusted risk of invasive epithelial ovarian cancer, according to endometriosis status**

	Patients with endometriosis (***n*** = 5945)	Controls (***n*** = 23780)
Number of patients with EOC	39	36
Incidence per 10,000 person-years	11.64	2.66
Crude HR (95% CI)	4.48 (2.84-7.06)^*^	1.00
Adjusted HR (95% CI)	5.62 (3.46-9.14)^*^	1.00

EOC: invasive epithelial ovarian cancer; HR: hazard ratio; 95% CI: 95% confidence interval.

Adjusted for pelvic inflammatory disease, infertility status, cardiovascular disease, diabetes mellitus, chronic liver disease, and rheumatic disease.

*P < 0.001.

In an effort to clarify the role of age in the relationship between endometriosis and EOC, we performed subgroup analysis based on age, using 4 age groups (those <30, 30–39, 40–49, and ≥50 years). The median age of the women with and without endometriosis who had a diagnosis of EOC was 43.6 and 43.3 years, respectively, which was without statistical significance (*p* =0.8783). The risks of EOC in the women with endometriosis increased with increasing age. The EOC IR of the women with endometriosis ranged from the lowest IR of 4.99 per 10,000 person-years at age <30 years to the highest IR of 35.81 at age ≥50 years (Table 3). Using the youngest group (women <30 years) as the reference, the HRs (95% CI) of the women with endometriosis aged 30–39, 40–49, and ≥50 years were 1.74 (95% CI 0.38-7.96), 1.87 (95% CI 0.43-8.01), and 5.46 (95% CI 1.18-25.32), respectively, in the crude model (*p* =0.0223). After adjustment for confounders, the adjusted HRs (95% CI) of women with endometriosis aged 30–39, 40–49, and ≥50 years were 1.66 (95% CI 0.36-7.61), 1.70 (95% CI 0.38-7.59), and 4.97 (95% CI 1.03-24.09), respectively (*p* =0.0351). Both analyses revealed a risk of EOC in the women with endometriosis that significantly increased with age (Table 3).

**Table 3:** **An increased risk of epithelial ovarian cancer in women with endometriosis with age**

	Age < 30 years	Age 30-39 years	Age 40-49 years	Age ≥ 50 years	P*
	n = 573	n = 1791	n = 3023	n = 558	
IR	4.99	9.89	10.66	35.81	
Crude HR (95% CI)	1.00 (Reference)	1.74 (0.38-7.96)	1.87 (0.43-8.05)	5.46 (1.18-25.32)	0.0223
P**		0.4729	0.4022	0.0302	
Adjusted HR (95% CI)	1.00 (Reference)	1.66 (0.36-7.61)	1.70 (0.38-7.59)	4.97 (1.03-24.09)	0.0351
P**		0.5154	0.4853	0.0465	

IR: incidence rate (incidence per 10,000 person-years); HR: hazard ratio; *P: comparison among all groups. **p: comparison between study group and reference group (age < 30 years).

The risk of EOC in the women without endometriosis also increased with age (IR of EOC in this population ranged from 1.78 to 5.80 per 10,000 person-years in all age groups), except those women aged between 30 and 39 years--it decreased in this group. In spite of an increased EOC IR with increasing age in the women without endometriosis, the women with endometriosis still had a more progressively increased risk of EOC with increasing age than the women without endometriosis (crude HRs ranging from 2.80 to 6.74 and adjusted HRs ranging from 3.34 to 9.63) (Table 4). Both the crude (*p* =0.0015) and adjusted (*p* =0.0039) Cox models were statistically significant. Our results suggested that age was an important risk factor for EOC in women with endometriosis, since older women with endometriosis not only had the absolute highest risk of EOC (35.81 per 10,000 person-years of EOC IR), but also had a more significantly increased risk of EOC than younger women with endometriosis, with a HR of 4.97 (95% CI 1.03-24.09).

**Table 4:** **Incidence and crude and adjusted risk of invasive epithelial ovarian cancer, according to age**

	Age < 30 years (n =3148)		Age 30-39 years (n = 9310)		Age 40-49 years (n = 13747)		Age ≥ 50 years (n = 3520)	
	Patients	Controls	Patients	Controls	Patients	Controls	Patients	Controls
Diagnosis of EOC								
Yes	2	3	10	4	18	22	9	7
No	571	2572	1781	7515	3005	10702	549	2955
IR	4.99	1.78	9.89	0.96	10.66	3.38	35.81	5.80
Crude HR (95% CI)	2.80 (0.47-16.74)	1.00	13.80 (3.80-50.11)^**^	1.00	3.03 (1.62-5.64)^*^	1.00	6.74 (2.51-18.10)^*^	1.00
Adjusted HR (95% CI)	3.34 (0.54-20.60)	1.00	19.41 (5.02-75.10)^**^	1.00	3.41 (1.76-6.61)^*^	1.00	9.63 (3.27-28.37)^**^	1.00

Patients: women with endometriosis; Controls: women without endometriosis; EOC: epithelial ovarian cancer; IR: incidence rate (incidence per 10,000 person-years); HR: hazard ratio; CI: confidence interval; *P < 0.001, **P < 0.0001.

**Figure 2:**
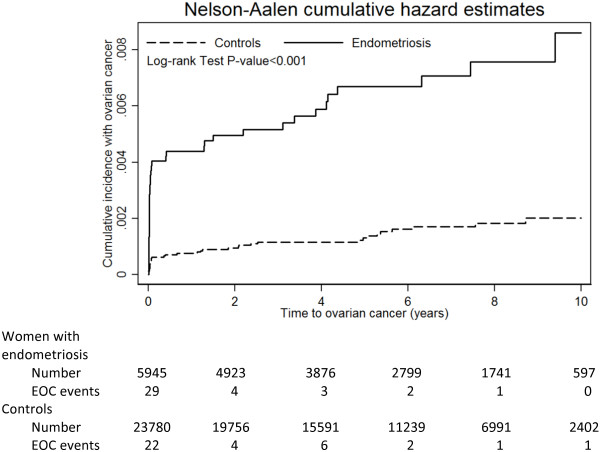
**The relationship between enrollment in this cohort and the occurrence of epithelial ovarian cancer.**

The role of follow-up time between enrollment and the occurrence of EOC (interval) was investigated. The median interval between the cohort index date and the date of a surgico-pathological diagnosis of EOC for the women with and without endometriosis was 505 days (ranging from 5 to 3433 days) and 824 days (ranging from 1 to 4095 days), respectively. There was a statistically significant difference between the 2 groups (*p* =0.0396), suggesting that women with endometriosis had a statistically significantly shorter interval in which to get EOC than women without endometriosis did. In fact, the highest risk of EOC was found in the first-year follow-up. The increased risk of EOC in the women with endometriosis remained consistent and persistent, since they had a significantly higher risk of EOC than the women without during the subsequent follow-up period (≥ one-year follow-up), with an adjusted HR of 3.32 (95% CI 1.48-6.68), after excluding the EOC cases in the first year of follow-up. All of this suggested that the women with endometriosis really did have a higher risk of EOC than the women without (Figure 2).

The median follow-up time for the women with and without endometriosis was similar, and without a statistically significant difference (2059 days, ranging from 3 to 4019 days vs. 2080 days, ranging from 1 to 5243 days, respectively, *p* =0.2267). Furthermore, there was no statistically significant difference in the median visits to gynecologists between the 2 groups (8.9, ranging from 6 to 77 visits vs. 9.1, ranging from 6 to 77 visits, for the women with and without endometriosis, respectively, *p* =0.6881). We separated the follow-up intervals into one and 2 years to test the IR of EOC in each group, and found that the EOC IR of the women with endometriosis was always higher than that of the women without, contributing to a consistently and persistently higher risk of EOC in the women with endometriosis than that in the women without, regardless of the interval of follow-up (*p* =1.000).

Finally, we investigated whether the increased risk of EOC in women with endometriosis might be influenced by the significantly increasing incidence of “endometriosis-associated EOC”, such as endometrioid and clear-cell subtypes [34–37]. As expected, women with endometriosis had a higher risk of “endometriosis-associated” EOC (adjusted HR 3.70, 95% CI 1.62-8.46), and the risk of clear-cell subtypes was much increased (adjusted HR 7.36, 95% CI 1.91-28.33). By contrast, other cell-type EOCs might not be associated with endometriosis, since there was no statistically significant difference between the women with and without endometriosis (adjusted HR 0.948, 95% CI 0.27-3.28).

## Discussion

Although many studies have supported the positive association between endometriosis and EOC, many uncertainties remain. One is the very low risk of developing EOC from endometriosis, estimated at a <1.5% lifetime probability, compared with 1% in the general female population [38]. In addition, co-morbidities of endometriosis, such as primary infertility, seemed more likely to carry a risk of EOC [20]. Many infertility studies have shown no association between endometriosis and EOC [18, 21]. Furthermore, contraception methods (tubal ligation, oral pills, etc.), especially the use of oral pills, which might be prescribed for treating symptoms of endometriosis, are inversely related to the risk of EOC [14, 39]. Finally, hysterectomy and/or bilateral salpingo-oophorectomy might have a protective effect on the occurrence of EOC [23, 35]. The strength of this study was that surgical confounders, such as tubal ligation, hysterectomy, and bilateral salpingo-oophorectomy were well controlled, because they had been excluded in this study.

This study has another important strength, that is, it might be the first nationwide, population-based study in an Asian country. One study was conducted in Japan, but it might not be representative of the general population of women with “endometriosis” in Asia [1]. In the Japan study [1], the diagnosis of “endometriosis” was made by ultrasound, the disease was limited to the ovary, and only 1/3 of cases had surgical confirmation. In our study, all subjects with endometriosis were found in an administrative data set, and all of the women had a new surgico-pathological confirmation, including nearly half who were diagnosed as having ovarian endometrioma (data not shown). Buis and colleagues found that the increased risk was especially high in women with pathologically-confirmed endometriosis after subfertility treatment, with an HR of 12.4 (95% CI 2.8-54.2), compared with an HR of 8.2 (95% CI 3.1-21.6) in women whose endometriosis was diagnosed by self-report or medical record information at subfertility treatment and/or in the nationwide pathology database [22], suggesting that a bias of selected subjects for either the study group or the control group might significantly influence the risk estimation. Although the strict criterion of “a surgico-pathological diagnosis” of endometriosis might exaggerate the incidence rate of EOC in the endometriosis arm of the study, with a subsequently inflated risk of EOC in this arm, the risk of EOC in women with endometriosis in the current study was still far lower than in Buis and colleagues’ report (HR 5.62 vs. HR 12.4). In addition, Melin and colleagues [11] showed that women who underwent unilateral oophorectomy for endometriosis or radical surgical excision of all visible endometriosis had a significantly reduced risk of later development of EOC, with adjusted odds ratios of 0.19 (95% CI, 0.08–0.46) and 0.30 (95% CI, 0.12–0.74), respectively, compared with controls. In our current study, we did not analyze the effect of different surgical procedures in the management of endometriosis, and we believe that some of the women may have been treated with unilateral salpingo-oophorectomy or radical surgical excision, which might be a protective procedure against the occurrence of EOC, but we still found that those women with a new surgico-pathological diagnosis of endometriosis had a persistently and consistently higher risk of later development of EOC during the follow up in the current study. Finally, after adjustment of confounding factors, the women with endometriosis had a much higher risk of EOC than the women without (adjusted HR 5.62, 95% CI 3.46-9.14 vs. crude HR 4.48, 95% CI 2.84-7.06), suggesting that women with a new surgico-pathological diagnosis of endometriosis indeed have a higher risk of EOC than women without.

The other argument, that the highest proportion of EOC was diagnosed in the first year of follow-up, including 29 of the 39 EOCs in the women with a new surgico-pathological diagnosis of endometriosis and 22 of the 36 EOCs in the women without, contributing to an unusually high IR of EOC in both groups, was raised. Furthermore, the some of the women had a diagnosis of EOC immediately within the enrolment period. Twenty-four and fifteen patients with EOC were found in the women with and without a new surgico-pathological diagnosis of endometriosis, respectively (*p* =0.0853), if we defined that the enrolment period was less than 90 days. An additional argument was that all women with endometriosis had a surgery, but not all of the controls did. It is possible that the women underwent surgery for a clinical diagnosis of endometriosis but were found to have cancer, resulting in more EOC patients in the endometriosis arm being detected. The number of cases of endometriosis was a quarter of cases in the women without endometriosis and every change by one case in the incidence (cancer) of women with a new surgico-pathological diagnosis of endometriosis would exaggerate the incidence ratio fourfold. All of this emphasized the potential risk of biases secondary to surveillance, including surgery, symptoms, and frequency of gynecologist/obstetrician visits in our current study. They are worthy of being discussed. However, our goal was to determine the risk of EOC due to endometriosis in Taiwanese women. Therefore, a surgico-pathological procedure, which is a “gold-standard” tool to diagnose endometriosis [40], should be performed. The IR of EOC in the women either with or without endometriosis was relatively evenly distributed in their own group, and the EOC IR of the women with endometriosis was always higher than that of the women without, contributing to a consistently and persistently higher risk of EOC in the women with endometriosis than in the women without, regardless of the interval of follow-up (*p* =1.000). In addition, after excluding EOC cases during the first-year follow-up, the median time between enrolment and occurrence of EOC in both groups was similar, without a statistically significant difference (1296 vs. 1402 days, *p* =0.8979), suggesting that the increased risk of EOC in women with a new surgico-pathological diagnosis of endometriosis may be irrelevant to surgery. All this suggested that endometriosis itself had a strong association with EOC, not only for a synchronous tumor (EOC arising from endometriosis), but also for any newly developed EOC.

Along with the findings of Kobayashi [1] and Pearce [16], our results supported the lack of an association between follow-up time and risk of EOC. There was no statistically significant difference in the frequency of visits to gynecologists between women with and without endometriosis during the follow-up period (8.9 vs. 9.1 visits, *p* =0.6881), which further supported the likelihood that the increased risk of EOC in women with endometriosis might not be biased by surveillance. Our above-mentioned results did not support the findings in Melin’s [9] and Brinton’s reports [4, 7] showing the existence of an increased risk of EOC as follow-up time increased (HR of 1.43 increased to 2.23) [9].

Investigating the contribution of age to the association between endometriosis and EOC was another of this study’s strengths. The risk of EOC was found to increase with the increase in age at diagnosis of ovarian endometrioma [1]. Women above 50 years of age had a higher HR of 13.2 (95% CI 6.90-20.9) [1]; Melin and colleagues had a similar finding [9]. Our study findings supported these previously published data. The absolute EOC IR of women with endometriosis consistently and persistently increased with increasing age (from the lowest IR of 4.99 per 10,000 person-years in women aged <30 years to the highest IR of 35.81 person-years in women aged ≥50 years). Using women with endometriosis <30 years old as a reference to perform risk estimation, we found that women with endometriosis aged ≥50 years had a statistically significantly increased risk of EOC (adjust HR 4.97, 95% CI 1.03-24.09, *p* =0.0465).

There were some limitations in our current study. First, we excluded women who had endometriosis diagnosed before the year 2000, and of the most importance, only women who had a surgico-pathological diagnosis of endometriosis were enrolled into the study. Therefore, some women who may have had endometriosis were excluded from this study, due to the absence of a surgico-pathological confirmation. In addition, as shown above, these women may have undergone unilateral salpingo-oophorectomy or radical surgical excision of all visible endometriosis, which might have decreased the risk of EOC [11]. Second, we excluded women with a possibly high or low risk of EOC. For example, women who never visited a gynecologist were excluded in our original design.

Third, we did not evaluate how many women regardless of endometriosis status had visited physicians for antenatal check-up or for pill refills during the study period. In addition, we did not investigate the yes/no and time course of the use of oral pills, which might have had a protective effect against the occurrence of EOC, and the protection might have been stronger in those women with long-term use [14, 39]. It is reasonable to suppose that women with endometriosis might have a more pronounced trend and longer interval in the use of pills for symptom control [41]. However, we used the following strategies, including (1) the exclusion of women without a gynecologist visit; and (2) a 1:4 match by obstetric history and frequency of gynecological/obstetric providers’ outpatient visits. Both might minimize the potential biases of surveillance or the frequency of medical care. We believed that the frequency of antenatal check-up and/or health consultation would be similar between both groups.

Fourth, we did not investigate the main symptoms of the women seeking gynecologic services. Some of them might have presented with persistent symptoms, which might overestimate the risk. And, some of them might have already had a co-existing EOC, which might further overestimate the risk of EOC in women with endometriosis. The inherent main symptoms of the women with and without endometriosis visiting gynecologists might be different. For example, women with endometriosis might frequently have complaints of dysmenorrhea and abdominal pain. Our data also showed the significant difference in baseline characteristics in both groups. The women with endometriosis had higher rates of comorbid PID, infertility, CVD, DM, CLD, and RD than the women without (Table 1). However, after adjustment of all confounders, the adjusted HR was even higher than the crude HR. Finally, the EOC IR of women without endometriosis was similar to that of the database of the National Cancer Registration System [31]. All of this supported the existence of an actual risk of developing EOC among women with a new surgico-pathological diagnosis of endometriosis.

## Conclusions

Although many uncertainties could not be totally avoided in our current study, our findings corroborate a previously reported association between endometriosis and an increased risk of EOC. The risk of EOC in women with a new surgico-pathological diagnosis of endometriosis was consistently and persistently higher than that in women without endometriosis, regardless of age and follow-up period, and the risk was particularly high in the older population (≥50 years). This population group was apparently at risk of EOC because of their higher incidence rate in absolute terms and comparably higher risk of EOC than that of age-matched women without endometriosis and/or younger women with endometriosis. Active and intensive surgical intervention should be considered with these older women with endometriosis if they do not have other contraindications for surgery.
